# Marine virus predation by non-host organisms

**DOI:** 10.1038/s41598-020-61691-y

**Published:** 2020-03-23

**Authors:** Jennifer E. Welsh, Peter Steenhuis, Karlos Ribeiro de Moraes, Jaap van der Meer, David W. Thieltges, Corina P. D. Brussaard

**Affiliations:** 1 Department of Coastal Systems, NIOZ Royal Netherlands Institute for Sea Research, and Utrecht University, P.O. Box 59, 1790 AB Den Burg, Texel, The Netherlands; 2 Department of Marine Microbiology & Biogeochemistry, NIOZ Royal Netherlands Institute for Sea Research, and Utrecht University, P.O. Box 59, 1790 AB Den Burg, Texel, The Netherlands

**Keywords:** Parasitology, Viral transmission, Microbial ecology, Marine biology

## Abstract

Viruses are the most abundant biological entities in marine environments, however, despite its potential ecological implications, little is known about virus removal by ambient non-host organisms. Here, we examined the effects of a variety of non-host organisms on the removal of viruses. The marine algal virus PgV-07T (infective to *Phaeocystis globosa*) can be discriminated from bacteriophages using flow cytometry, facilitating its use as a representative model system. Of all the non-host organisms tested, anemones, polychaete larvae, sea squirts, crabs, cockles, oysters and sponges significantly reduced viral abundance. The latter four species reduced viral abundance the most, by 90, 43, 12 and 98% over 24 h, respectively. Breadcrumb sponges instantly removed viruses at high rates (176 mL h^−1^ g tissue dry wt^−1^) which continued over an extended period of time. The variety of non-host organisms capable of reducing viral abundance highlights that viral loss by ambient organisms is an overlooked avenue of viral ecology. Moreover, our finding that temperate sponges have the huge potential for constant and effective removal of viruses from the water column demonstrates that natural viral loss has, thus far, been underestimated.

## Introduction

**Figure 1:**
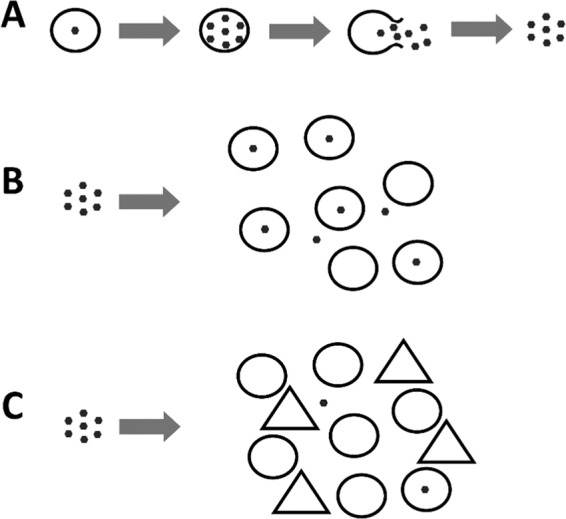
This conceptual diagram shows lytic viruses (⬣) infecting and replicating inside their susceptible host (○). After the host lyses, virus progeny are released into the surrounding environment where they have the potential to proceed and infect a new host (**A**). In a simple system, these newly produced viruses are available to infect the available succeeding hosts (**B**). In complex systems, typically found in nature, viruses may be lost due to interactions with non-host organisms (△), resulting in reduced encounter rates and disease prevalence within the ecosystem (**C**).

Viruses are the most numerically abundant entities in the oceans with an estimated abundance up to 10^8^ mL^−1 ^^1^. Via infection and mortality of their microorganism hosts, viruses have the ability to regulate not only host population dynamics but also to drive biogeochemical cycling and carbon sequestration within marine systems^2–6^. As lytic virus infections of microbial hosts inevitably result in the death of the host cell, any decay of infectious virus particles is likely to have important repercussions for hosts as it results in lower encounter rates and thus reduced infection levels. Despite marine viruses influencing fundamental biological processes^2,3^, research investigating virus decay (e.g. loss of infectivity) and particle loss has primarily focused on abiotic factors such as UV radiation and temperature (loss of infectivity) and adhesion to clay particles and aggregates (loss of viral particles)^7–11^. However, biological factors resulting in removal of virus particles from the water column is still understudied (Fig. 1). Marine heterotrophic nanoflagellates have been reported to graze on viruses, albeit at a relatively low rate of 0.1% of the viral population day ^−1^, or a clearance rate of around 4%^12,13^. Furthermore, the Red Sea sponge *Negombata magnifica* was reported to filter viruses with an average efficiency of 23%^14^. And recently the appendicularian stage of the pelagic *Oikopleura dioica* tunicate was reported to remove viruses at significant rates^15^.

While such reductive effects of non-host organisms on marine viral abundance are still understudied, similar removal effects have been well demonstrated in other aquatic host-pathogen systems. For instance, infective stages of helminths are removed by a wide range of non-host organisms via predation and other mechanisms^16–20^, with reductions in free-living infective stages reducing infection levels in downstream hosts^14^. The variety of non-host organisms causing the reductions is not only limited to active predators but also includes passive predators such as filter feeders and organisms creating physical barriers between the parasite and its host^16,21^. Thus, non-host organisms are known to affect parasite transmission in macro parasite-host systems, a phenomenon known as transmission interference^17,22–24^. In this study, we used various ecologically relevant non-host organisms to assess their effects on transmission interference on a microparasite-host system. Specifically, we investigated the potential of a variety of pelagic and benthic marine non-host species, including bivalve filter feeders and decapod predators, to remove a marine algal virus from the surrounding seawater. We used the marine algal virus PgV, which is host specific and known to infect the bloom forming algae, *Phaeocystis globosa*^25^, and a selection of non-host organisms that are found in coastal areas where the algal host,virus, and non-host organisms coincide. In addition, the organism most proficient in reducing viral abundance was studied in more detail to determine if efficient removal of viruses could be sustained for a prolonged time. Understanding which organisms and to what extent non-host organisms regulate marine viral abundances is unreservedly important for more accurate predictions of the ecological impact viruses have on host population dynamics in the seas and oceans.

## Materials and methods

### Viruses and non-host organisms

The marine phytoplankton virus PgV-07T (NIOZ culture collection^25^) was chosen as a model system because it can be clearly discriminated (and enumerated) from co-occurring bacteriophages using flow cytometry, based on fluorescence after staining with the nucleic acid-specific dye SYBR Green I^26^. *Phaeocystis globosa* strain G (A) (culture collection of the University of Groningen, the Netherlands) is the host of the double stranded DNA virus PgV-07T (hereafter known as PgV; around 150 nm diameter and 470 nm kbp genome size^25^) and is an ecologically important primary producer with a wide distribution in temperate (coastal) seas during spring and summer^9,27–31^. Algal cultures were grown in Mix-TX medium^32^ at 15 °C and a light:dark cycle of 16:8 h. Cultures were transferred weekly to keep the cells growing exponentially. The virus PgV was produced by infecting exponentially growing hosts and allowing complete lysis to occur during the following week. Prior to experimental use, the lysate was cleared from most of the lysed *P. globosa* cell debris by centrifugation (Eppendorf 5810 R, Hamburg, Germany) at 2450 × g for 30 min at 15 °C. The supernatant containing the PgV was carefully pipetted off and stored at 15 °C until experimental use (max. 2 days). Filtration of the supernatant resulted in reduced PgV abundances in the filtrate and hence we chose to use unfiltered supernatant for our experiments. The infectivity of the PgV batches used for the experiments was determined by most probable number (MPN) endpoint dilution^33^. Percentage infectious viruses (obtained by dividing the MPN abundance by the total abundance from flow cytometric analysis^26^) was near 100%, ensuring that potential removal of PgV by non-host organisms was not a selective process for either infectious or non-infectious virus particles.

PgV was enumerated according to the protocol by Brussaard *et al*. (2004)^26^ with modification as described by Mojica *et al*. 2014^34^). In short, stored samples collected during the experiment were thawed and diluted in sterile 0.2 µm filtered TE buffer (10:1 Tris-EDTA, pH 8.2; Minisart high flow Syringe Filter, Sartorius A.G., Göttingen, Germany), stained with the nucleic acid-specific green fluorescent dye SYBR Green I (Invitrogen-Molecular Probes) for 10 min in the dark at 80 °C, after which PgV was enumerated using a BD FACSCanto™ flow cytometer (BD Biosciences, USA). The trigger was set on the green fluorescence for the detection of stained PgVs. Data were processed using FCS Express 4 software (De Novo Software).

Non-host organisms tested for their ability to reduce viral abundance were chosen based on their geographic distribution coinciding with that of the algal host-virus model system and included organisms of varying feeding mechanisms (filter feeders, predators, etc.) as well as range in size (small copepods to large oysters). Bivalves such as oysters, mussels and cockles are found in intertidal-subtidal areas and are considered bioengineers, altering substrate type, filtering vast amounts of water and removing particles <250 µm in size^35^. Organisms such sponges, anemones and sea squirts are found on hard surfaces such as harbor walls and are typically subtidal but can also be found in some intertidal areas. Both sponges and sea squirts are capable of filtering large volumes of water (300 L h^−1^ and 200 mL min^−1^ respectively^36,37^) and retaining nano-sized particles^14,38–40^. Polychaete larvae and copepods are pelagic, feeding on prey <53 µm in size^41^. Copepods live in coastal and upwelling regions and switch prey between algae and ciliates under 60 µm in size with clearance rates of <86 m1 d^−l^)^42,43^. The species used, i.e. anemones (*Actinia equina*), barnacles (*Semibalanus balanoides*, attached to one valve of an empty mussel shell), cockles (*Cerastoderma edule*), crabs (*Carcinus maenas*), mussels (*Mytilus edulis*), oysters (*Magallana gigas*), sea squirts (*Styela clava*) and adult copepods (*Acartia tonsa*, >125 µm) and polychaete larvae (a mix of species, >125 µm) were all collected between spring and summer from the coastal area along the island of Texel (the Netherlands). Breadcrumb sponges (*Halichondria panicea*) for the first experiment (Exp. 1) were collected along the same coast, but to prevent impacting the local sponge community too much the sponges for subsequent tests were collected from the Oosterschelde (southern Netherlands) and transported to the NIOZ in cool boxes. After collection, all organisms were gently cleaned to remove any visible epibionts. Before being transferred to sterile 100 mL polystyrene pots for the experiments, the cleaned organisms were starved for 24 h in flow-through aquaria (80 × 40 × 40 cm) at 15 °C with a light:dark cycle of 8:16 h.

## Experimental set-up

### Experiment 1: Removal of viruses when in the presence of non-host organisms

Experiment 1 (Exp. 1) assessed a variety of organisms for their ability to interfere with a marine virus-host transmission pathway by removing infectious virus particles from the water. Sterilized 100 mL polystyrene pots with screw cap (VWR International, Leuven, Belgium) were used during the experiment as aquaria. Prior to the experiment the pots were sterilized using 6 M HCl for 1 h, followed by rinsing in deionized water and then finally washed in 90 °C deionized water to remove any traces of HCl. The experiment consisted of two types of treatment: the first with the non-host organism (typically 1 individual per pot, except for barnacles which were 10 on an empty mussel valve, and copepods and polychaete larvae which had 16 individuals per pot) in 80 mL of the PgV lysate (around 1 × 10^6^ mL^−1^), and the second treatment with only the lysate to assess for adherence to the pots. In addition, an extra control with only the test organism in culture media was used to assess for the introduction of viruses by the test organism. As these controls indicated that no viral particles in the same size range as PgV were introduced, they were left out of further analysis. Each of the two regular treatments was replicated six times. All trials took place in a single climate room kept at 15 °C. During the experiment, samples (1 mL) were taken using sterile pipettes (one for each replicate) and placed into 1.5 mL Eppendorf tubes containing 20 µL 25% glutaraldehyde (0.5% final concentration, EM-grade, Sigma-Aldrich, St. Louis, USA) for 30 min at 4 °C, flash-frozen in liquid nitrogen and stored at −80 °C until further analysis (Brussaard *et al*. 2004). Samples were taken before the test organisms were added (pre-test or PT), 15 min after the animal were adapted to the pot (T0), after a period of 3 h (T3) and then after 24 h (T24), with the exception of crab, cockle, barnacle and mussel treatments where samples were taken before the test organisms were added (pre-test or PT) and after a period of 3 h (T3).

### Experiment 2: Continuous clearance of virus by breadcrumb sponge

Breadcrumb sponges were shown to significantly decrease PgV abundance (Exp. 1) and so Experiment 2 (Exp. 2) was designed to assess the ability of the sponges to continuously remove PgV over a longer time period. The experimental set-up was similar to Exp. 1, but PgV (approx. 2 × 10^6^ mL^−1^) was added (‘spiked’) at 20 min intervals for 6 h to avoid depletion of viruses by the sponges. Samples (1 mL) were taken using individual sterile pipettes prior to and immediately after adding viruses to the system to allow for the calculation of clearance rates. Treatments consisted of test organism spiked with PgV, and two controls (sponge spiked with growth medium to test for disturbance effect due to spiking, and PgV, spiked with PgV to obtain the ultimate PgV abundance without the sponge present). All treatments were replicated four times but due to the mortality of one sponge during the experiment, only three replicates were used for statistical analysis. Samples were processed and stored as in Exp. 1.

### Statistics

All statistical tests were carried out using R (R Core Team 2014). For Exp. 1, the effect of the presence of non-host organisms on changes in PgV abundance over time, was statistically tested by comparing changes in PgV abundance from one sampling time to the next, between the treatment where a non-host organism was present and the control treatment. A univariate analysis of variance (ANOVA) was used when samples were only taken at two time points (i.e., when there was only a single change observed between the start and end of the trial), whereas a multivariate analysis of variance (MANOVA) was used when samples were taken at more than two occasions (e.g. at the start, after 15 min, 3 h and then again after 24 h). The univariate test statistic is the F-value, the multivariate test statistic is Pillai’s trace. Significances indicate that viral abundances over time differed among treatments. As there are only two treatments (with and without non-host organisms), the tests are in fact equivalent to the t-test and to Hotelling’s T^2^ test.

For Exp. 2, sponge clearance rates were calculated for 20 min intervals using the viral abundances directly before spiking and the viral abundance immediately after the aquaria were spiked with viruses. Sponge clearance rates were determined using the following formula:

$${C}_{l}=(V/Kt)ln({N}_{0}/{N}_{t})$$

whereby *C*_l_ is volume of water cleared of suspended particles per unit of time; *V* is the volume, *K* is the number of individual sponges and *N*_0_ and *N*_t_ are the virus cell abundance at time 0 and time t, respectively^44^. In this study one piece of sponge (i.e. one individual of 52 mm Ø in size; 1.76 ± 0.99 g dry weight; 0.42 ± 0.16 g ash free dry weight) was used per treatment. Clearance rates were calculated for the period after clearance had stabilized, i.e. from 1–5.5 h into the experiment, unless otherwise stated. Linear regressions were subsequently used to test for density dependency by comparing the viral abundances directly before spiking against clearance rates for that specific 20 min period.

## Results and Discussion

### Experiment 1: Removal of viruses by non-host organisms

**Figure 2:**
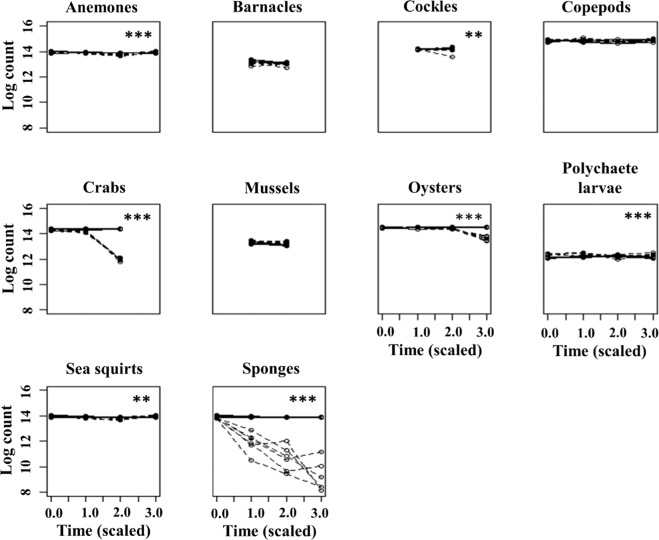
Viral abundance (log PgV mL) over time in the presence (dashed lines) and absence (control; black lines) of a non-host organisms. Asterisks identify non-host organism which had a significant effect on changes in viral abundance (‘***’ P < 0.001 ‘**’P < 0.01 ‘*’P < 0.05). Each line represents one replicate. Note that time axis is not continuous, but scaled to four observation times, start of experiment (time 0), approximately 15 min (time 1), 3 h (time 2), and 24 h (time 3) after the start. The y-axis shows the Log of PgV counts, that is the natural log base of the exponential of the virus counts. All virus counts were in the range of x10^6^.

Ten marine non-host species were tested to assess their ability to reduce abundances of the model virus PgV. As expected, the control treatments containing only PgV showed no significant decline in PgVs over time and controls containing only the non-host organisms did not display virus enrichment. All non-host organisms, except barnacles, copepods and mussels, significantly affected PgV abundances (Table 1, Fig. 2), showing that the interference of virus transmission by non-host organism is a common process. Whilst anemones, polychaete larvae and sea squirts tested as significant, the rate of change was very small so that they did not result in an ecologically relevant reduction in viruses by the end of the experiment (Supplement Fig. S1). Conversely, another sessile marine tunicate or ‘sea squirt’ has been reported to remove up to 7 × 10^5^ *Emiliania huxleyi* viruses mL^−1^ (by 0.4 animals mL^−1^), with a clearance rate of 50 mL ind^−1^ day^−1 ^^15^ suggesting that removal of viruses by tunicates may be species-specific. The presence of oysters and crabs resulted in significant reduction of PgV abundances over the 24 h experimental period, i.e. <12% and 90% respectively within 3 h. In other studies, after exposure to water containing human enteric viruses, bivalves and crabs showed internal accumulation of the viruses, with recovery of viruses from tissues such as the digestive tract^45–49^. This indicates that both decapods and bivalves have the potential to take up viruses from their surrounding environment and, collectively, significantly contribute to the reduction in viral abundances. Moreover, studies have shown that after uptake by bivalve species viral particles released via fecal matter are inactive and thus suggesting that the digestion process renders the particles non-infective^50,51^. In other marine macroparasite-host systems crabs and oysters have been shown to effectively reduce the number of free-living trematode parasites^16,18^. While the infective cercarial stages of trematode parasites are considerably larger (body length of several hundred µm^52^) than virus particles, our study demonstrates the extent of their interactions and their potential to alter a wide range of infectious diseases within a system. Besides the existing studies focusing on the concentration of viruses and their impact on the human food chain we are, to our knowledge, the first to report the removal of non-human viruses by marine shellfish.

**Table 1:** Uni- (ANOVA) and multivariate (MANOVA) analyses results testing for the effect of the presence of a non-host organism on changes in PgV abundance over time, compared to the control treatment where no non-host organisms were present.

Test organism	Pillai trace	- F	*P*	Significance
Anemones	0.84	—	<0.001	***
Barnacles	—	0.0	0.970	
Cockles	—	0.0	0.997	
Copepods	0.16	—	0.457	
Crabs	0.99	—	<0.001	***
Mussels	—	4.0	0.073	(*)
Oysters	0.93	—	<0.001	***
Polychaete larvae	0.79	—	<0.001	***
Sea squirts	0.71	—	0.004	**
Sponges	0.87	—	<0.001	***

Significance levels indicate whether there was a significant change in viral abundance (over time) between the control and treatments.

ANOVA tests used F values and were conducted when samples were only taken at two time points. MANOVA tests, however, were used when samples were taken at more than two time points and used Pillai’s trace test.

Significance codes are as follows: *** =0.001; ** =0.01; * =0.05; and (*) =0.1.

The strongest removal of PgV was caused by the sponges, not only by removing the most viruses but also because removal of viruses from the system began instantly (Fig. 2, Supplement Fig. 1). The Breadcrumb sponge reduced PgV abundance by 94% in the first 3 h and a minimum of 14-fold by the end of the 24 h experimental period, with an end reduction of 98%. Sponges are known to be effective at filtering out small particles such as algal and bacterial cells^53–56^, as well as dissolved organic matter (DOM)^57^. According to the standard practical classification and operational definition, particles <0.2 µm are included in the DOM pool, i.e. the DOM particle size range also includes the nanometer range of virus particles. In this study we used only algal virus PgV-07T but, given the size range of prey sponges can take up, we would expect comparable removal rates of other viruses (algal viruses and bacteriophages). Natural seawater viruses, dominated by typically smaller-sized bacteriophages (20–60 nm range)^58^, have been reported to be removed by a tropical sponge^14^. Furthermore, even the very small influenza viruses can be effectively removed by bivalves^51^. While not the focus of this study, the overall removal of virus particles is likely to have selected consequences for nutrient cycling via the direct loss of viruses (containing and average of 41 C and 16 N and 4.2 P atoms per virus capsid head; potentially accounting for <0.01 to 50% of the total marine DOP, <7% DON depending on the viral group and other physical parameters^59^) and the indirect alterations in biogeochemical cycling resulting from host cell lysis^2,3^. The effective removal of ecologically relevant virus concentrations by the temperate breadcrumb sponge highlights that the filtration activity and the removal of viruses from the surrounding water by non-host organisms is compelling. The virus removal rates shown here are much higher than previously reported for other sponge species. For example the tropical sponge *Negombata magnifica* reduced viruses with an efficiency of 23 ± 3%^14^. Discrepancies in sponge efficiency between their and the present study may be a result of anatomical differences between the sponges used; here we used a temperate sponge from the order Halichondrida, whereas Hadas and coworkers (2006) used a tropical sponge from the order ‘Poecilosclerida’. Furthermore, the sponge species used in our study has fluctuating removal efficiencies depending on the season. This study was conducted during the period when the sponges exhibit their highest energy demand (April and August^60^). Importantly, the differences between the studies illustrate that the contribution of sponges in controlling viral abundance in marine systems is thus far substantially underestimated. Such high reductions in viral abundance are likely to have ecological consequences with knock-on effects for the algal host and virus-host contact rates.

### Experiment 2: Continuous clearance of viruses by breadcrumb sponge

**Figure 3:**
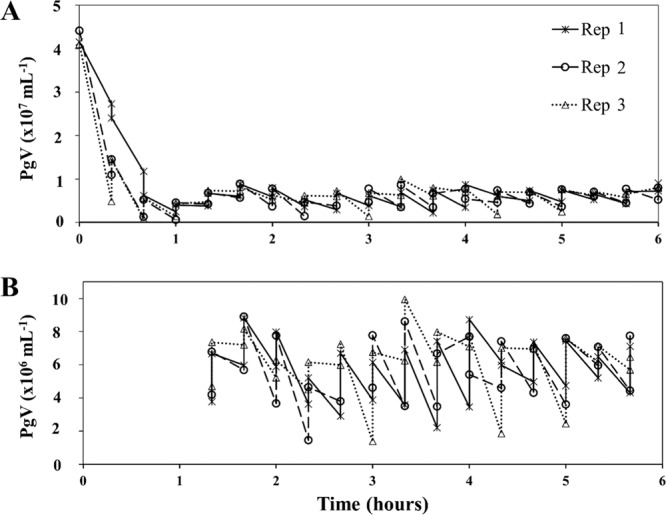
The removal of PgV by breadcrumb sponges over the entire incubation period (**A**) and a close up of the stabilized period from 1 to 5.5 h into the experiment when PgV was added at 20 min intervals (**B**). Experiment was performed in triplicate (Rep 1–3).

To test the sponges’ ability to consistently remove viruses over time, we spiked replicates with PgV every 20 min for up to 6 h with approx. 2.5 × 10^6^ PgV mL^−1^ to avoid complete depletion in the surrounding water (Fig. 3). Removal rates of the 3 sponges (one sponge died during the experiment) in the first 3 h and over 24 h were comparable to exp. 1 with a 91% and 94% reduction in viruses. After 1 h the removal of PgV stabilized and the sponge continued to clear PgV at a rate of around 5 mL h^−1^ (Fig. S2). During the stabilized period (1 to 5.5 h into the experiment) the sponges removed a total of about 9.3 × 10^7^ PgVs (Fig. 3). Initial PgV abundances varied between the two experiments but all initial abundances were within the natural range found in marine environments^61^. Our results thus demonstrate a constant and very effective virus removal by the breadcrumb sponge. Clearance rates of the viruses by the breadcrumb sponges stabilized after the start of the experiment and remained constant until the end of the testing period. Sponges cleared viruses at a rate of 176 mL h^−1^ g tissue dry wt^−1^ (based on initial 15 min). Given that virus clearance rates reported thus far (e.g. Hadas *et al*. 2006^14^; 648 mL h^−1^ g tissue wet weight converted to 38 mL h^−1^ g tissue dry wt^−1^ as outlined in Frost 1978^62^) are lower than in our study, the ecological importance of virus loss by sponge activity is most likely extremely underestimated. Previous studies on viral and DOM loss by sponges have primarily used tropical sponge species^55,63^. To our knowledge, we are the first to show that a temperate sponge species is efficient at removing viruses and thus it is likely that other temperate sponge species are also able to reduce viral abundance with equal or better efficiency.

Theoretically there might have been the possibility that sponges filtered the suspension volume (80 mL) more than once, however given that the mean clearance rate was 176 mL h^−1^ g sponge dry weight and the average sponge dry weight per replicate was 1.76 ± 0.99 g, the average clearance rate was 77 mL (range between 34 and 121 mL) within the 15 min sampling period. Although a higher time resolution is recommended it was logistically constrained by the sample processing time required immediately after taking the samples. By utilizing a controlled experimental design and setup such as the one used in this study we are able to show that the loss of viruses was a direct effect of the sponge and not an artefact due to interactions caused by the presence of other organisms within the system. Furthermore, unlike Hadas *et al*. 2006^14^, by using a controlled experiment and filtered water we can also conclude that there was no other potential ‘food’ in the water, potentially supporting previous suggestions that viruses act as a nutrient source and a part of the sponge pump^57,64^.

Reductions in natural virus abundance by such high degree as shown in our study, have the potential to impact local microbial host population dynamics as the virus-host contact rate drops due to the decline in virus abundance. For PgV specifically, viruses infecting *P. globosa* have been shown to contribute considerably to bloom demise and, depending on the environmental conditions, viral activity may even prevent bloom formation^8,65,66^. Thus any reduction in contact rate between PgV and its *P. globosa* host results in reduced virally induced mortality of the algal host, subsequently promoting a longer blooming period or shifting the share of loss factors from viral lysis to grazing^67,68^. Sponges have a crude ability to remove particles based on size^69–73^ and it is well documented that bacteria and phytoplankton are a major part of sponge prey^70,74,75^. This could influence host population dynamics directly (removal of host algae) and indirectly (removal of viruses). This complex form of transmission interference may thus reduce contact rates between viruses and their hosts even further (due to the dual reduction of host and virus). Therefore, the presence of a non-host organism may be a ‘double edged sword’ affecting the abundance of both viruses and hosts. We conducted a pilot experiment to compare simultaneous removal of PgV, *P. globosa* and bacteria, and, indeed, all three types of particles were efficiently cleared (within 24 h) by the breadcrumb sponge. The bacterial clearance rate of 535 mL h^−1^ g tissue dry wt^−1^ falls within the rates published for other sponges (10–5000 mL h^−1^ g tissue dry wt^−1^)^55,62,63,69,76^. The *P. globosa* clearance rate (68.2 mL h^−1^ g tissue dry wt^−1^) was in the middle range of published rates for phytoplankton^76^. Substantial variation in clearance rates for both phytoplankton and bacteria do occur which may be caused by factors such as initial particle concentration, time of year, temperature, species and individual sponge behavior^63,76,77^. Regardless of these differences, our study demonstrates that the virus clearance rates are within the range of the acknowledged natural food particles, bacterial and algal cells.

Given that sponges can be found in high densities in coastal regions, such as harbors^78^, as well as in tropical^79^ and deep sea reefs^80,81^, collectively they are highly likely to continuously interact with the viruses in the water column creating strong localized differences in viral abundance. For example, the overall effects of sponges on viruses here in the Netherlands is likely to have less of an impact than in other regions such as coral reefs or harbors where the majority of surfaces comprise of hard substrate. In such locations sponge coverage can be high, e.g. ranging from 45–70 m^−2^ (Indonesia^82^) to>1400 individuals m^−2^ (Ireland^83^), therefore, in locations such as relatively enclosed bays, the effect of sponges on virus removal is probably vastly underestimated. For example, in a hypothetical hard-substrate bay measuring 10 km × 10 km × 0.01 km, an average sponge density of 1,400 sponges m^−2^, and a sponge clearance rate of viruses of 5 mL h^−1^ (Exp 2 this study), an estimated 7 × 10^9^ L h^−1^ is cleared of viruses by the sponges. That equates to 1.7% of the bays water volume every hour. Given stable conditions (e.g. stagnant water), all the viruses in the bay could, hypothetically, be cleared within 3 days. Although such estimates should be approached with caution, the above estimation underlines the enormous potential of sponges as virus loss factor whereby the ultimate ecological impact depends on the local conditions such as tide phase, current strength, local sponge cover, other non-host organisms potentially removing viruses from the water column (e.g. bivalves), as well as host presence and abundance.

In conclusion, our results stress the notion that a wide range of non-host organisms and in particular sponges, have the potential to reduce nano-sized pathogen abundance via transmission interference. It is very likely that there are many more species capable of removing viruses from the water column. It is also likely that there are a variety of factors which facilitate or impede the removal of viruses, such as stratification or mixing of the water column altering contact rates, ambient temperatures affecting non-host organism feeding rates, free-floating clays and aggregates to which viruses may adhere^8,84–86^ all of which should be tested for their effects on transmission interference. An interesting follow up experiment would be to expand on the experiments investigating the effect of sponges on the contact rates of host and virus when both are present. Considering that even temperate sponges effectively remove viruses from the water column (this study), the global ecological consequences are expected to be considerable and hence, there is a need for further investigations to determine how, when and to what extent non-host organisms affect virus-host dynamics.

## Supplementary information

Supplementary material.

## Data Availability

The datasets generated during and/or analysed during the current study are available from the corresponding author and are available via the 4TU data repository [10.4121/uuid:9dbe319e-c42e-4b47-a4ce-5d2e33243c1c].
