# Supplementary material for: Marine virus predation by non-host organisms
Source: Sci Rep. 2020 Mar 23;10:5221. doi: 10.1038/s41598-020-61691-y (PMC7089979; doi:10.1038/s41598-020-61691-y)
Supplement: Supplementary file 1 — Supplementary material. [file 41598_2020_61691_MOESM1_ESM.docx]

**Marine virus predation by non-host organisms**

Jennifer E. Welsh^1*^, Peter Steenhuis^2^, Karlos Ribeiro de Moraes^2^, Jaap van der Meer^1^, David W. Thieltges^1^, Corina P.D. Brussaard^2^

**Supplementary material.**

**Experiment 1: Removal of viruses in the presence of non-host organisms.**


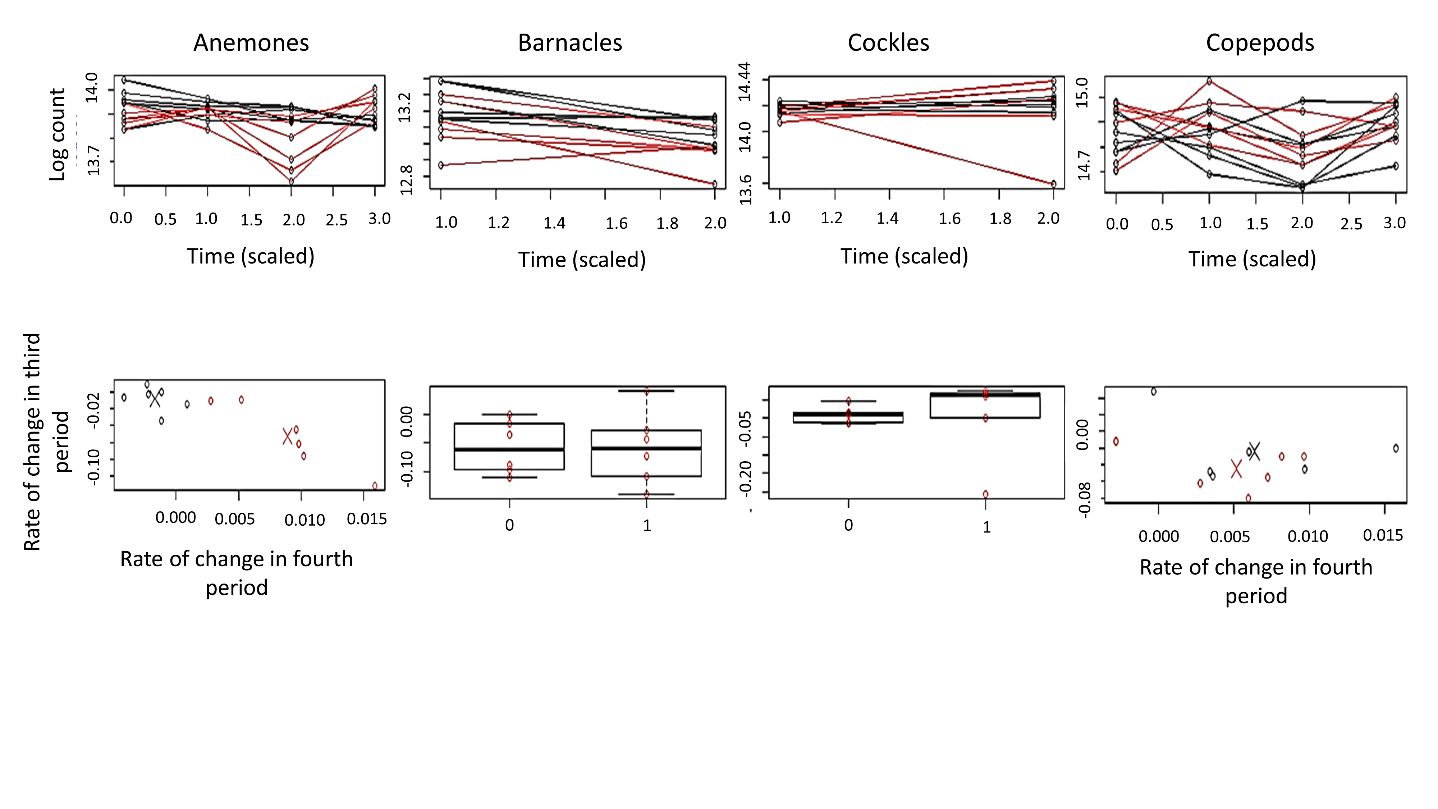


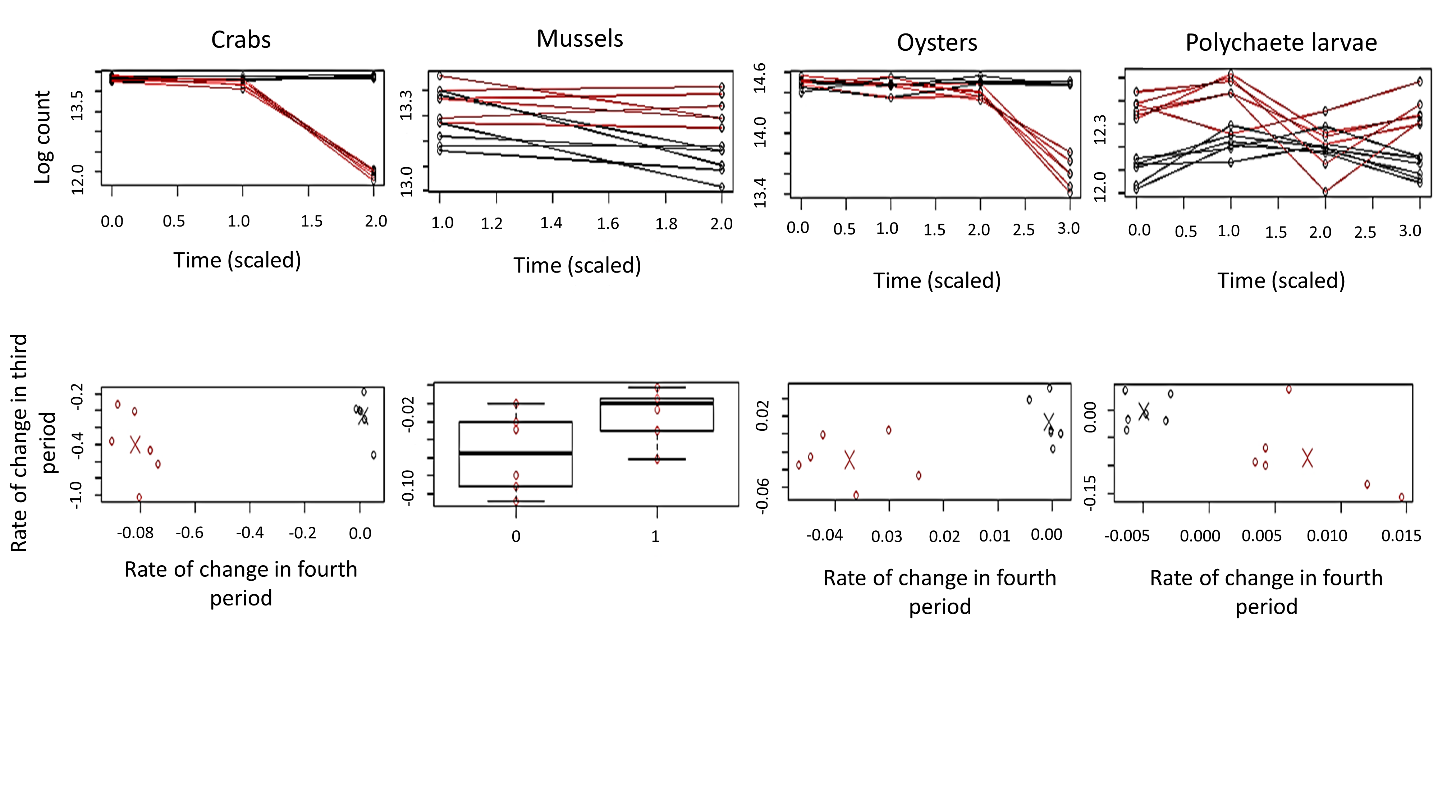


Figure S1. Viral log counts and rates of change in viral abundance over time for individual replicates (red lines) and for each test organism as well as controls (viruses only, black lines). Rate of change over the fourth period amongst control replicates (black circles) and test organism replicates (red circles) and their means (crosses in associated colour) is shown as negative or a reduction in viral abundance when the rate of change is >0.00. Anemones, crabs, oysters, polychaete larvae, sea squirts and sponges all significantly affected viral abundance (see main text). Whilst anemones, polychaete larvae and sea squirts tested as significant factors in viral abundance, from the above graphs it is evident that they do not have an ecologically relevant influence on overall viral abundance. Crabs, oysters, and sponges, however, clearly significantly reduce viral abundance by the end of the experiment. Sponges reduced viral abundance at a rapid rate from the outset, clearing almost all of the viruses by the end of the experiment.


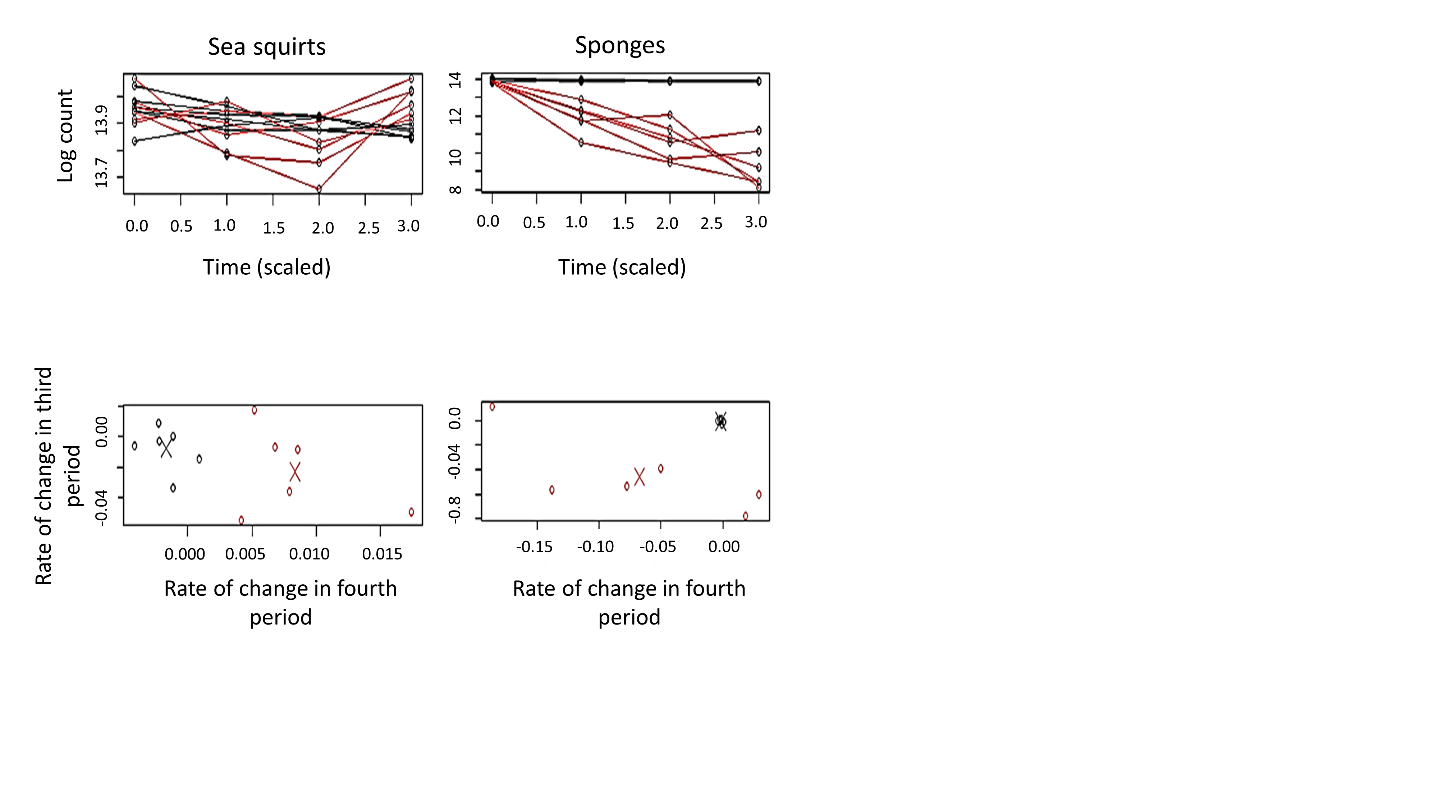


Figure S1. Viral log counts continued.

**Experiment 2: : Continuous clearance of virus by breadcrumb sponge**

**
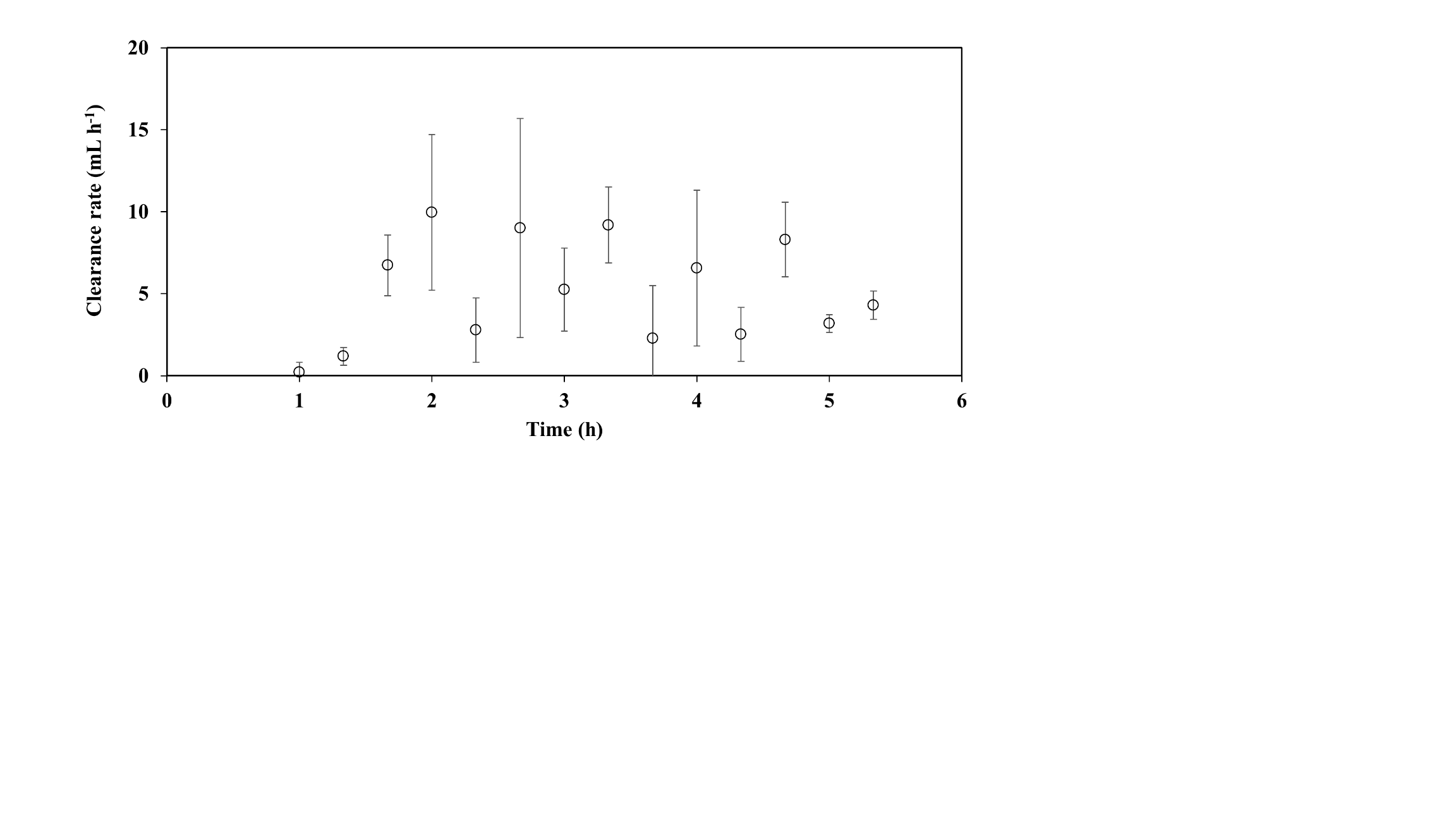
**

Figure S2. Mean clearance rates (± standard error) of PgV by breadcrumb sponges during the stable clearance period of Exp. 2.
